# Assessment of *Tamarindus indica* Extracts for Antibacterial Activity

**DOI:** 10.3390/ijms12106385

**Published:** 2011-09-26

**Authors:** Uchechukwu U. Nwodo, Grace E. Obiiyeke, Vincent N. Chigor, Anthony I. Okoh

**Affiliations:** 1 Applied and Environmental Microbiology Research Group (AEMREG), Department of Biochemistry and Microbiology, University of Fort Hare, Private Bag X1314, Alice 5700, South Africa; E-Mails: vchigor@ufh.ac.za (V.N.C.); aokoh@ufh.ac.za (A.I.O.); 2 Department of Botany, Delta State University, Abraka, 330106, Delta State, Nigeria; E-Mail: ekygee@yahoo.com

**Keywords:** Tamarind, *Tamarindus indica*, extracts, antibacterial activity

## Abstract

Ethanolic and aqueous (hot and cold) extracts of the fruit pulp, stem bark and leaves of *Tamarindus indica* were evaluated for antibacterial activity, *in vitro*, against 13 Gram negative and 5 Gram positive bacterial strains using agar well diffusion and macro broth dilution techniques, simultaneously. The fruit pulp extracts exhibited a wide spectrum of activity; the cold water extract against 95.5% of the test bacterial strains; and the hot water and ethanolic extracts against 90.9% and 86.4%, respectively. In contrast the cold water extract of the leaves and stem bark, each was active against 16.7%; while the ethanolic extract of each was active against 75% of the test strains. The minimum inhibitory concentrations (MIC) ranged from 7.81 mg/mL against *Bacillus subtilis* ATCC 6051 to 31.25 mg/mL against *Escherichia coli* ATCC 11775; and the minimum bactericidal concentration (MBC) ranged from 125 mg/mL against *Pseudomonas aeruginosa* ATCC 10145 to 250 mg/mL against *Bacillus subtilis* ATCC 6051.

## 1. Introduction

All through history, irrespective of culture, plants have been a dependable source of medicine [1,2]; and 70–90% of the world’s rural population still depends on herbal remedies for health care [3]. *Tamarindus indica* L., (Tamarind), family, Leguminosae, is one such widely used medicinal plant. It is found in virtually all tropical climatic regions, from India through Africa to the Caribbean and South America and up to Southern Florida. Its uses are as varied as the cultures that use it. It is often more difficult to determine which use is more important, as food and beverage [4,5] or as folklore medicine [5,6]. In the West African sub-region, including Nigeria, it is widely used as both food and medicine. The pulp has been documented in both the British and American pharmacopoeias as anti-pyretic, antiscorbutic, laxative, carminative and remedy for biliousness and bile disorder [5–8]; and the leaves have antihelmintic and vermifuge properties, destroying intestinal parasites [6]. The work reported here was carried out to validate the medicinal use of this plant in Northern Nigerian folklore.

## 2. Results and Discussion

### 2.1. Results

Generally, the cold water extracts gave higher percentage yields (w/w) after extraction, range, 9.7% (stem bark) to 14.4% (fruit pulp); while ethanol had the least yield, 8.8% to 9.6%. Likewise the fruit pulp gave the highest yield, 9.6% to 14.4%. The pH ranged from 2.0 for the cold water extract of the fruit pulp to 5.5 for the cold water extract of the leaves (Table 1).

Carbohydrates, reducing sugars, tannins and saponins were detected in all extracts. With the exception of the cold and hot water extracts of the leaves, flavonoids and Cyanogenic glycosides were present in all extracts. Anthroquinone was detected in cold water extract of the fruit pulp in addition to all the ethanolic extracts. Alkaloids were present in all the ethanolic extracts as well as the cold and hot water extracts of the fruit pulp. Steroles were not found in any extract and terpenes occurred only in the ethanolic extract of the fruit pulp (Table 2).

**Figure 1:**
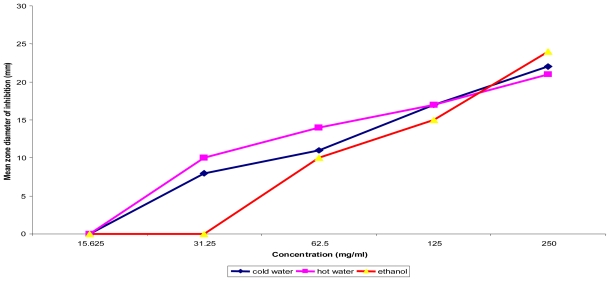
Concentration dependent assay of *T. indica* fruit pulp on *Ps. aeruginosa* ATCC 10145.

**Figure 2:**
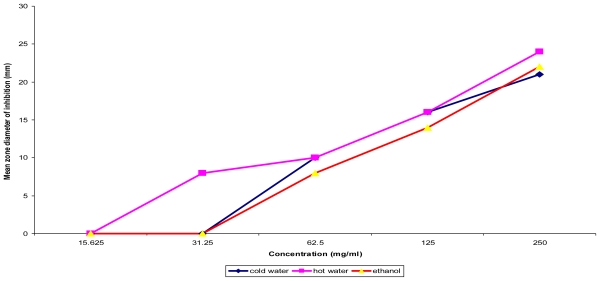
Concentration dependent assay of *T. indica* fruit pulp on *Bacillus subtilis* ATCC 6051.

The cold water extract of the fruit pulp was active against all (100%) of the non diarrheagenic bacterial strains tested achieving inhibition zone diameters (IZDs) ranging from 18 ± 0.0 mm to 24.5 ± 0.71 mm and minimum bactericidal concentration (MBC) of 125 mg/mL (Tables 3 and 4); but both the hot water and ethanolic extracts were active against 6 (85.71%) each. Similarly, both ethanolic extracts of the leaves and stem bark showed activity against 5 (71.43%) of the non diarrheagenic bacterial strains each whilst the cold water extract of leaves, stem bark and hot water extract of the stem bark each showed activity against 28.57%, respectively. The fruit pulp extracts were active against all five Gram positive test bacterial strains with MBC values of 125–250 mg/mL; but the ethanolic extracts of the stem bark (SET) and leaves (LET) showed activity against 80% each (Table 3). Seven (7) local clinical isolates of *E. coli* from infantile diarrhea (numbered 1–7) and 3 of *Pseudomonas aeruginosa* (numbered 1 and 2, respectively) including one found to be multiple drug resistant (coded MDR) were specifically tested with SET and all the fruit pulp extracts and the results are shown in Table 5. With the exception of one isolate, *E. coli* 2, which showed no susceptibility to all the extracts tested and *E. coli* 4, which was not affected by FET, the local isolates including the multiple drug resistant *P. aeruginosa* were susceptible with IZD range of 10.50 ± 0.00 mm to 28.00 ± 0.00 mm. Figures 1 and 2 shows the dose response curves of *T. indica* fruit pulp extract tested *in vitro* against representatives of Gram negative and Gram positive bacterial strains, respectively. The result showed that the IZD increased directly with the concentrations of the extracts used irrespective of the solvent used for extraction.

### 2.2. Discussion

In all cases the highest yield was obtained with cold water extraction, followed by hot water and ethanol the least signifying that most of the components extracted were water soluble. Relatively, more yield was obtained from the fruit pulp with every extraction solvent than from leaves or stem bark showing that more components were contained in the fruits. It is interesting that the fruit which is frequently consumed as food or beverage contained large quantities of water soluble constituents, some of which were shown in this work to have antibacterial activity. What needs to be ascertained is whether the antibacterial activity would remain *in vivo* after they have been acted upon by the digestive enzymes. The yield obtained may be limited by the method of extraction, maceration, which has been noted to be inferior to Soxhlet extraction technique [9]. The low pH of the extracts may reflect the presence of high levels of oxalic acid, ascorbic acid and, particularly, tartaric acid which is an unusual plant acid [5].

The phytochemical constituents detected, including flavonoids, alkaloids, tannins, cyanogenic glycosides and anthroquinones. These may have accounted for antibacterial activity [10,11]. These phytochemicals and some other aromatic secondary metabolites have been suggested to serve as natural agents that protect plants agents against microbial pathogens and insect predators [12]. Their distribution varied more with plant part (leaves, stem bark and fruit pulp) than with solvent of extraction in contrast to the observation of Doughari [13]. The uneven distribution of these constituents in plant parts reflects the natural functions of these parts as manufacturing organs (the leaves), storage organs (the fruit) or as avenues of excretion of wastes (stem bark). This may explain why there was concentration of the antibacterial activity in the fruit pulp and the stem bark rather than the leaves. For the leaves and stem bark, antibacterial activity was found almost exclusively in the ethanol extracts, implying either that the active principles were principally alcohol soluble or that they were stabilized by the alcohol. However, considering that similar aqueous extracts of the fruit pulp were even more active than the ethanolic extracts, it is likely that the differences in activity observed between the aqueous and ethanolic extracts of the leaves reflect differences in the types of compounds extracted. Demonstration of antibacterial activity against both Gram negative and Gram positive bacteria signify a broad spectrum of activity by the extract tested; but it is not certain that this may be interpreted to mean broad spectrum of activity for the specific active principle(s) contained in the extract since partial purification experiments have shown that a crude extract contains several components some of which may interact additively or synergistically to produce a broad spectrum effect. The scope of this work did not permit investigation into the component(s) containing the active compounds. Water and alcohol are the most common media for preparation of herbal concoctions by the herbalists; and extracts prepared with the same solvents in this work showed remarkable antibacterial activity, thus authenticating the medicinal value of these in folklore practices. The dose response effect *in vitro* shows that the IZD could be used to estimate the level of activity of each extract. Acidity as a mechanism of the antibacterial effect of each extract is ruled out because the *in vitro* medium for bacterial culture is buffered (pH 7.3 ± 0.1) and, therefore, the extracts were active at pH other than the low value determined for each and this concurs with the findings of Doughari [13].

## 3. Experimental Section

### 3.1. Plant Materials

The fruits, leaves and stem bark of *T. indica* were obtained from Sokoto South Local Government Area, Sokoto State of Nigeria. The plant was identified taxonomically and voucher specimen deposited at the Herbarium of the Department of Botany, University of Nigeria, Nsukka.

### 3.2. Preparation of Plant Extract

Fresh leaves and stem bark of *Tamarindus indica* were rinsed thoroughly in running tap water, chopped to tiny pieces and air dried at room temperature for a period of 14 days; and subsequently pulverised with a mechanical grinder. The flesh or pulp covering the seeds was also removed and dried as above. Approximately 50.0 g of ground leaves, stem bark and fruit pulp were each macerated in 200 mL of cold water and absolute ethanol (BDH) for a period of 24 h at room temperature. The hot water extraction of each of the three plant parts was as described by Okoli *et al*. [14]. Each preparation was filtered through a Whatman No. 1 filter paper and filtrate evaporated to dryness in a steady air current after which all extracts were stored in a sterile container and stored at room temperature.

### 3.3. Phytochemical Analysis

All the extracts obtained were screened for the presence of alkaloids, saponins, tannins, anthraquinones, glycosides, flavonoids, reducing sugar, carbohydrates and sterols using the methods of Trease and Evans [15] and Harbone [9] and are as follows;

#### 3.3.1. Test for Carbohydrates

Few drops of Molisch’s reagent were added to an aqueous solution of each extract followed by vigorous shaking. Thereafter, 1.0 mL of conc. H_2_SO_4_ was added carefully by sliding down the walls of the tube gently to form two layers. The solution was examined for the appearance of brown ring separating the solution into two layers.

#### 3.3.2. Test for Reducing Sugar

To 1.0 mL of aqueous solution of each extract was added 3.0 mL of a mixture of equal volumes of Fehling’s solutions I and II and boiled in a water bath at about 40 °C for 2 min. A brick red color at the bottom of the test tube was an indication of the presence of reducing sugar.

#### 3.3.3. Test for Glycosides

Tests for glycosides were performed as follows:

- To 0.1 g of each extract in a test tube was added 5.0 mL of water and the mixture heated in a water bath at 100 °C for 2 min. The mixture was filtered through a Whatman No. 1 filter paper. A mixture of Fehling’s solutions I and II were added to the filtrate until it became alkaline: followed by heating for 2 min;
- The above procedure was repeated, except that 5.0 mL of dilute sulphuric acid was added to 0.1g of the extract instead of water: and the quantity of precipitate formed was noted;
- About 0.1 g of each extract was put into a stoppered conical flash in which was suspended a strip of sodium picrate paper. The flask was warmed gently for about an hour at 37 °C and allowed to stand. The test paper was examined for any change in color.

#### 3.3.4. Test for Tannins

Approximately 0.1 g of each extract was added to 2 mL of distilled water and boiled gently for 2 min. It was then filtered while hot, and allowed to cool. Ferric chloride solution (5%) was added drop-wise and the experiment observed for color change.

#### 3.3.5. Test for Saponins

Presence of saponins was determined by their frothing property as well as capacity to form emulsion with oils.

- For the frothing test, about 5 mg of extract was shaken vigorously with and examined for frothing;
- For the emulsification test, 2 drops of olive oil was added to 5.0 mL of aqueous solution of the extract in a test, shaken vigorously and observed for formation of an emulsion. The control was without extract but water and olive oil.

#### 3.3.6. Test for Flavonoids

A 5.0 g weight of the extract was detanned with acetone; the residue was extracted in warm water after evaporating the acetone in a water bath. The mixture was filtered and the filtrate used for the following tests:

- *Lead Acetate Test*: To 2.0 mL of the detanned aqueous solution was added 10% Lead acetate solution; a colored precipitate indicates the presence of flavonoids;
- *Ferric Chloride Test*: A 2.0 mL volume of detanned aqueous suspension of extract was diluted with distilled water in a ratio of 1:4 and a few drops of 10% ferric chloride solution added. A green or blue solution indicates the presence of flavonoids.

#### 3.3.7. Test for Anthroquinones

Approximately 0.1 g of the extract was mixed with 5.0 mL of chloroform and agitated for 5.0 min. The solution was filtered and equal volume of ammonia was added to the filtrate and agitated again. A brick red color in the upper aqueous layer indicates the presence of free anthroquinones.

#### 3.3.8. Test for Terpenes and Sterols

A 1.0 g weight of the extract was mixed with 5.0 mL of 95% ethanol and then filtered. The filtrate was evaporated to dryness and the residue re-dissolved in 5.0 mL of anhydrous chloroform and then filtered. The latter filtrate was divided into two portions for the following tests:

- *Liebermann-Burchard Test*: The first portion was mixed with 1 mL of acetic anhydride followed by the addition of 1.0 mL of concentrated Sulfuric acid gently down the side of the test tube to form a layer underneath. The formation of a reddish violet color at the junction of the two liquids and a green color in the chloroform layer would indicate the presence of terpenes;
- *Salowski’s Test:* To the second portion of the solution was added 2.0 mL of concentrated Sulfuric acid carefully down the side of the tube so that the sulfuric acid formed a layer. A reddish brown color at the interface would indicate the presence sterols.

### 3.4. Test Bacterial Strains

Clinical and type cultures were used for the studies, the clinical strains include *Staphylococcus aureus* from a case of non-gonococcal urethritis, 7 strains of *Escherichia coli* from diarrheal stools of infants and 3 antibiotic resistant strains of *Pseudomonas aeruginosa*. The type cultures are 2 strains of *Bacillus cereus* (NRRL 14724 and NRRL 14725) obtained from the Department of Microbiology, University of Nigeria, Nsukka; *Pseudomonas aeruginosa* (ATCC 10145), *E. coli* (ATCC 11775), *B. subtilis* (ATCC 6051), and *Staph. aureus* (ATCC 12600) obtained from Bioresources Development and Conservation Project (BDCP), Nsukka; and *Salmonella kintambo* (SSRL 113) provided by the Department of Veterinary Microbiology and Pathology, University of Nigeria, Nsukka. Each test bacterial strain was purified by re-isolating three successive times on Mueller Hinton agar (Oxoid) and identity reaffirmed by standard bacteriological techniques [16]. Stock cultures were maintained in nutrient agar slants at 4 °C.

### 3.5. Screening Extracts for Antibacterial Activity

The extracts were screened for antibacterial activity using the agar well diffusion technique [17]. MacFarland nephelometry standardized inoculums of the test bacterial strain was adjusted to 1.0 × 10^6^ CFU/mL (Gram-positive bacteria) or 5 × 10^5^ CFU/mL (Gram-negative bacteria), [18]. A 100 μL volume of the bacterial suspension was spread over Muller Hinton agar (MHA) medium at room temperature before boring 6.0 mm (diameter) wells in the agar using a sterile cork borer. A 100 μL volume of extract, reconstituted in sterile distilled water to a concentration of 250 mg/mL, was introduced in triplicate wells into the MHA cultures. The plates were allowed to stand for about 1 h to allow diffusion to take place and then incubated at 37 °C for 24 h. The inhibition zone diameter was measures to the nearest mm.

### 3.6. Determination of Minimum Inhibitory Concentration (MIC) and Minimal Bactericidal Concentration (MBC) of Extracts

The Minimum Inhibitory Concentration (MIC) for each extract and test organism was determined by agar well diffusion method and by the macro-broth dilution technique [18]. A 125 mg/mL concentration of the reconstituted extract was serially diluted in two fold, down to 3.91 mg/mL. A 100 μL volume of each dilution was introduced into duplicate wells in the MHA plates pre-inoculated with test bacterial strain; and incubated at 37 °C for 24 h. The MIC was taken as the lowest concentration of the extract showing measurable inhibition zone.

For the macro-broth dilution technique, a 100 μL volume of each dilution of the extract was introduced into duplicate tubes of 2.0 mL Mueller Hinton broth (MHB) seeded with 100 μL of the standardized suspension of the test bacterial strain. Incubation was at 37 °C for 24 h; and MIC was taken as the lowest concentration of the extract that made the culture show no visible growth.

The Minimum Bactericidal Concentration (MBC) was determined using a modified agar well diffusion technique [18]. A 2 mm diameter agar disc cut out from the inhibition zone of the last three consecutive wells in each dilution showing inhibition was inoculated into a fresh sterile nutrient broth medium. The broth cultures were incubated at 37 °C for 24 h after which 100 μL was spread over a fresh sterile MHA. The MHA culture was in turn incubated at 37 °C for 24 h and the least concentration of the extract showing no growth was taken as the MBC. An MBC which coincided with or was next to the MIC value was considered bactericidal while those that differed markedly were considered bacteristatic [19].

## 4. Conclusions

*Tamarindus indica* is a medicinal herb that could be considered for integration into orthodox healthcare given that it is also commonly consumed as food or beverage and, therefore, generally regarded as safe (*gras*). In addition, the folkloric use of *T. indica* in the treatment of various ailments and enteric disturbances have been shown to hold true, empirically, in this research as high potency against pathogenic bacteria was obtained. The antibacterial activity exhibited by the ethanolic extract of the stem bark of *T. indica* was significant in various respects; but, the solvent of extraction and the plant part extracted was important in the activity shown by the plant. However, the fruit extracts showed better activity than the stem bark extracts which is the part used by herbal practitioners. Furthermore, the bacterial strains used for this work were those involved in enteric disturbances, food borne diseases and sexually transmitted infections which are amongst the most common diseases of concern in the tropics. Thus, this justifies the use of this herb in traditional practices for the treatment of ailments caused by these organisms and more.

## Figures and Tables

**Table 1:** The yield and pH of the various crude extracts of *Tamarindus indica.*

Extract	Yield (%)	pH
Leaves (L)		
Cold Water (LCW)	5.76 (11.5)	5.45
Hot Water (LHW)	5.21 (10.4)	4.99
Ethanol (LET)	4.38 (8.8)	4.71
Stem bark (S)		
Cold water (SCW)	4.85 (9.7)	4.81
Hot water (SHW)	4.65 (9.3)	4.70
Ethanol (SET)	4.58 (9.2)	4.62
Fruit pulp (F)		
Cold water (FCW)	7.21 (14.4)	2.00
Hot Water (FHW)	6.54 (13.1)	2.91
Ethanol (FET)	4.82 (9.6)	3.18

**Table 2:** Phyto-chemical Constituents of Extracts of *Tamarindus indica.*

Constituents tested	Presence of constituent in plant Extract								

	Leaves			Stem bark			Fruit pulp		

	LCW	LHW	LET	SCW	SHW	SET	FCW	FHW	FET
Carbohydrate	++	++	+	++	++	++	+	+++	+
Reducing sugar	++	+	+	++	+	+	+	++	+
Tannins	+	+	++	+	+	+	+	+	+
Flavonoids	ND	ND	+	+	+	+++	++	+	+++
Anthroquinone	ND	ND	+	ND	ND	++	+	ND	++
Saponins	+	+	++	++	++	+++	+++	+++	+++
Alkaloids	ND	ND	+	ND	ND	+++	++	+++	+++
Cyanogenic glycosides	ND	ND	+	+	+	++	++	++	++
Terpenes	ND	ND	ND	ND	ND	ND	ND	ND	+
Sterols	ND	ND	ND	ND	ND	ND	ND	ND	ND

Present in high mount (+++); present in moderate amount (++); present in low amount (+); not detected (ND); LCW = leaves cold water extract, LHW = leaves hot water extract, LET = leaves ethanol extract; SCW = stem cold water extract, SHW = stem hot water extract, SET = stem ethanol extract; FCW = fruit pulp cold water extract, FHW = fruit pulp hot water extract, FET = fruit pulp ethanol extract.

**Table 3:** Antibacterial activity of the various parts of *Tamarindus indica* against test bacterial isolates.

Bacterial Strain	Mean Inhibition Zone Diameter (250 mg/mL)									

	Leaves			Stem bark			Fruit pulp			Control

	LCW	LHW	LET	SCW	SHW	SET	FCW	FHW	FET	Ciproflox
*E. coli (clin)*	10.50 ± 0.25	0.00	8.0 ± 0.25	8.0 ± 0.0	13.0 ± 0.0	20.0 ± 1.41	20.0 ± 0.0	23.0 ± 0.0	18.0 ± 0.0	24.0 ± 0.45
*E. coli* ATCC 11775	0.00	0.00	10.0 ± 0.75	7.0 ± 0.0	7.0 ± 0.0	10.0 ± 0.0	20.0 ± 0.0	19.0 ± 0.0	10.0 ± 0.0	31.85 ± 0.25
*Salmonella typhi (clin)*	0.00	0.00	0.00	0.00	0.00	0.00	20.5 ± 0.71	20.0 ± 0.0	12.0 ± 0.0	19.0 ± 0.25
*Salmonella kintambo* SSRL 113	0.00	0.00	0.00	0.00	0.00	0.00	19.0 ± 0.0	19.0 ± 0.0	21.0 ± 0.0	24.8 ± 0.50
*Staph. aureus (clin)*	0.00	0.00	8.50 ± 0.25	0.00	0.00	19.5± 0.71	24.5 ± 0.71	12.0 ± 0.0	23.0 ± 0.0	25.85 ± 0.25
*Staph. aureus* ATCC 12600	0.00	0.00	0.00	0.00	0.00	0.00	18.0 ± 0.0	19.0 ± 0.0	14.0 ± 0.0	23.25 ± 0.25
*Ps. aeruginosa (clin)*	0.00	0.00	11.50 ± 0.75	0.00	0.00	23.0 ± 0.0	21.5 ± 0.71	17.0 ± 0.0	21.5 ± 0.71	23.25 ± 0.50
*Ps. aeruginosa* ATCC 10145	9.5 ± 0.25	0.00	11.50 ± 0.75	0.00	0.00	19.0 ± 0.0	21.5 ± 0.71	21.0 ± 0.0	23.0 ± 0.41	26.0 ± 0.71
*B. subtilis* ATCC 6051	0.00	0.00	10.50 ± 0.25	0.00	0.00	16.0 ± 0.0	20.5 ± 0.71	24.0 ± 0.0	18.5 ± 4.95	31.0 ± 0.25
*Proteus mirabilis (clin)*	0.00	0.00	10.50 ± 0.25	0.00	0.00	16.0± 1.41	20.5 ± 0.71	0.00	0.00	22.5 ± 0.71
*B. cereus* NRRL 14724	0.00	0.00	9.5 ± 0.69	0.00	0.00	18.0 ± 0.0	21.5 ± 0.71	17.0 ± 0.0	21.50 ± 0.71	24.25 ± 0.50
*B. cereus* NRRL 14725	0.00	0.00	12.50 ± 0.25	0.00	0.00	10.5±0.71	18.5 ± 0.71	15.0 ± 0.0	20.50 ± 0.71	26.0 ± 0.71

**Table 4:** The minimum inhibitory concentration (MIC), minimum bactericidal concentration and MIC-minimum bactericidal concentration (MBC) index on the test isolates.

Bacterial Strain	SET			FCW			FHW			FET		

	MIC	MBC	MBC-MIC INDEX	MIC	MBC	MBC-MIC INDEX	MIC	MBC	MBC-MIC INDEX	MIC	MBC	MBC-MIC INDEX
*E. coli*	15.63	125	0.125	31.25	125	0.25	31.25	125	0.25	62.50	125	0.50
*E. coli* ATCC 11775	31.25	125	0.25	31.25	125	0.25	62.50	125	0.50	125	125	1
*Salmonella typhi*	0	ND	ND	0	ND	ND	62.50	125	0.50	125	125	1
*Salmonella kintambo* SSRL 113	0	ND	ND	31.25	0	ND	31.25	125	0.25	62.50	125	0.50
*Staph. aureus*	15.63	125	0.125	31.25	125	0.25	125	125	1	62.50	125	0.50
*Staph. aureus* ATCC 12600	0	ND	ND	31.25	125	0.25	62.50	125	0.50	62.50	125	0.50
*Ps. aeruginosa*	15.63	250	0.125	7.81	250	0.0312	31.25	125	0.25	62.50	125	0.50
*Ps. aeruginosa* ATCC 10145	7.81	125	0.063	31.25	125	0.25	31.25	125	0.25	62.50	125	0.50
*B. subtilis* ATCC 6051	7.81	250	0.0312	62.50	250	0.25	31.25	125	0.25	62.50	125	0.50
*Proteus mirabilis*	7.81	0	ND	62.50	0	ND	0	0	ND	0	0	ND
*B. cereus* NRRL 14724	15.63	125	0.125	62.50	125	0.50	62.50	125	0.50	62.50	250	0.25
*B. cereus* NRRL 14725	62.50	125	0.50	62.50	125	0.50	62.50	250	0.25	62.50	125	0.50

ND = Not determined; 0 = absence of activity.

**Table 5:** Effects of *Tamarindus indica* crude extracts On *E. coli* isolates from infantile diarrhea, *Pseudomonas aeruginosa* isolates and its multi-drug resistant strain (*Pseudomonas aeruginosa* MDR).

Bacterial Strain	Mean Inhibition Zone Diameter (250 mg/mL)				

	Stem bark	Fruit pulp			Control

	SET	FCW	FHW	FET	Ciproflox (20 μg/mL)
*E. coli* 1	20.0 ± 0.0	21.0 ± 0.0	19.0 ± 0.0	20.50 ± 0.71	23.25 ± 0.25
*E. coli* 2	0.00	0.00	0.00	0.00	11.0 ± 0.50
*E. coli* 3	10.50 ± 0.0	25.0 ± 0.0	12.0 ± 0.0	24.50 ± 0.71	13.50 ± 0.50
*E. coli* 4	13.50 ± 0.71	23.0 ± 0.0	22.0 ± 0.0	0.00	23.85 ± 0.45
*E. coli* 5	17.50 ± 0.71	26.0 ± 0.0	23.0 ± 0.0	27.0 ± 0.0	25.85 ± 0.25
*E. coli* 6	21.50 ± 0.71	17.0 ± 0.0	19.50 ± 0.71	18.0 ± 0.0	28.0 ± 0.40
*E. coli* 7	16.50 ± 0.71	28.0 ± 0.0	19.50 ± 0.71	14.0 ± 0.0	19.50 ± 0.0
*Ps. aeruginosa* 1	20.50 ± 0.71	24.0 ± 0.0	20.0 ± 0.0	18.0 ± 1.41	24.85 ± 0.71
*Ps. aeruginosa* 2	17.50 ± 0.71	19.0 ± 0.0	19.0 ± 0.0	26.0 ± 0.0	26.0 ± 0.45
*Ps. aeruginosa* (MDR)	19.0 ± 0.0	21.0 ± 0.71	14.0 ± 0.0	19.0 ± 0.0	17.50 ± 0.60

## References

[1] Stockwell C (1988). Nature’s Pharmacy.

[2] Thomson WAR (1978). Medicines from the Earth.

[3] Lai PK, Roy J (2004). Antimicrobial and chemopreventive properties of herbs and spices. Curr. Med. Chem.

[4] Anon B (1986). The Useful Plants of India.

[5] Morton J (1987). Tamarind. Fruits of Warm Climates.

[6] Pamploma-Roger GD Encyclopaedia of Medicinal Plants Education and Health Library Madrid, Spain 1999 2536

[7] Iwu IM (1993). Handbook of African Medicinal Plants.

[8] Raimondi L, Lodovici M, Guglielmi F, Banchelli G, Ciuffi M, Boldrini E, Pirisino R (2003). The polysaccharide from Tamarindus indica (TS-polysaccharide) protects cultured corneal-derived cells (SIRC cells) from ultraviolet rays. J. Pharm. Pharmacol.

[9] Harbone JB (1998). Phytochemical Methods: A Guide in Modern Techniques of Plant Analysis.

[10] Watt JM, Breyer-Brandwijk MG (1967). Medicinal and Poisonous Plants of Southern and Eastern Africa.

[11] Leven MD, vanden-Berghe DA, Marten T, Villentmick A, Lomweas EC (1979). Screening higher plants for biological activity. Planta Med.

[12] Marjorie MC (1999). Plant products as antimicrobial agents. Clin. Microbiol. Rev.

[13] Doughari JH (2006). Antimicrobial Activity of *Tamarindus indica* Linn. Trop. J. Pharm. Res.

[14] Okoli AS, Okeke MI, Iroegbu CU, Ebo PO (2000). Antibacterial activity of *Harungana madagasceriensis* leaf extracts. Phytother. Res.

[15] Trease GE, Evans WC (1978). Phytochemistry: Introduction and General Methods. Pharmacognosy.

[16] Cheesbrough M (1984). Medical Laboratory Manual for Tropical Countries, Volume II Microbiology.

[17] Okeke MI, Iroegbu CU, Eze EN, Okoli AS, Esimone CO (2001). Evaluation of extracts of the root of *Landolphia owerrience* for antibacterial activity. J. Ethnopharmacol.

[18] National Committee for Clinical Laboratory Standards (NCCLS) (1993). Methods for Dilution in Antimicrobial Susceptibility Tests: Approved Standard.

[19] National Committee for Clinical Laboratory Standards (NCCLS) (1992). Methods for Determining Bactericidal Activity of Antimicrobial Agents: Tentative Guideline.

